# Supplementary material for: C-Terminal Extensions of Ku70 and Ku80 Differentially Influence DNA End Binding Properties
Source: Int J Mol Sci. 2020 Sep 14;21(18):6725. doi: 10.3390/ijms21186725 (PMC7555691; doi:10.3390/ijms21186725)
Supplement: Supplementary file 1 [file ijms-21-06725-s001.pdf]

C-terminal extensions of Ku70 and Ku80 differentially influence DNA end binding properties

Takabumi Inagawa[1], Thomas Wennink[1], Joyce H.G. Lebbink[1,2], Guido Keijzers[1], Bogdan I. Florea[1], Nicole S. Verkaik[1], and Dik C. van Gent[1]

Supplemental methods

Analysis of SPR sensorgrams. Sensorgrams of Ku binding to DNA could not be fit accurately by a 1:1 binding model (Figure S4, left panel). The reason for this is that a small fraction of the protein remained bound to the flow cell during the dissociation phase. This residual binding occurred in the reference flow cell, but was always a little more pronounced in the DNA-modified flow cells, resulting in a fraction remaining after reference channel subtraction and causing deviation form 1:1 binding.

In an attempt to get rid of this residual binding we implemented the following changes: increased flow rate to 100 μl; decreased association time from 120 to 60 and 30 seconds, added glycerol; changed surfactant P20 concentration; changed immobilization levels of DNA from 10 RU to 5 RU, 2 RU, and 50 RU; added NSB reducer, left out regeneration step (used long dissociation for removal of bound Ku); removed DTT; changed from Tris to Hepes buffer; used KCl instead of NaCl; used a different protein batch; and included a size exclusion chromatography step to remove small fraction of unfolded/aggregated protein from the sample immediately before analysis. None of the above resulted in a change in the level of residual Ku binding to the modified flow cell, indicating that the deviation from 1:1 binding behavior was not due to reaction conditions, the presence of slow binding/dissociating contaminant proteins, a conformational change in Ku to an extreme high-affinity state, Ku rebinding to DNA during the dissociation phase, or mass transfer limitations.

We identified two conditions that increased the level of residual binding: reducing the amount of streptavidin on the chip surface, used for immobilisation of the DNA, and aging of the chip surface (Figure S4, Right panel). This indicated that the Ku protein interacts with the dextran matric on the chip, but to a larger extend in the flow cells immobilized with DNA than on the reference flow cell. This might be due to the negatively charged DNA influencing the association rate constant for the interaction with the matrix. We took this into account by fitting data according to a two-state binding model that allows for this interaction to form in a DNA-dependent manner. Obtained affinity and rate constants were similar to those obtained from a 1:1 binding model (results not shown).

Supplementary figures

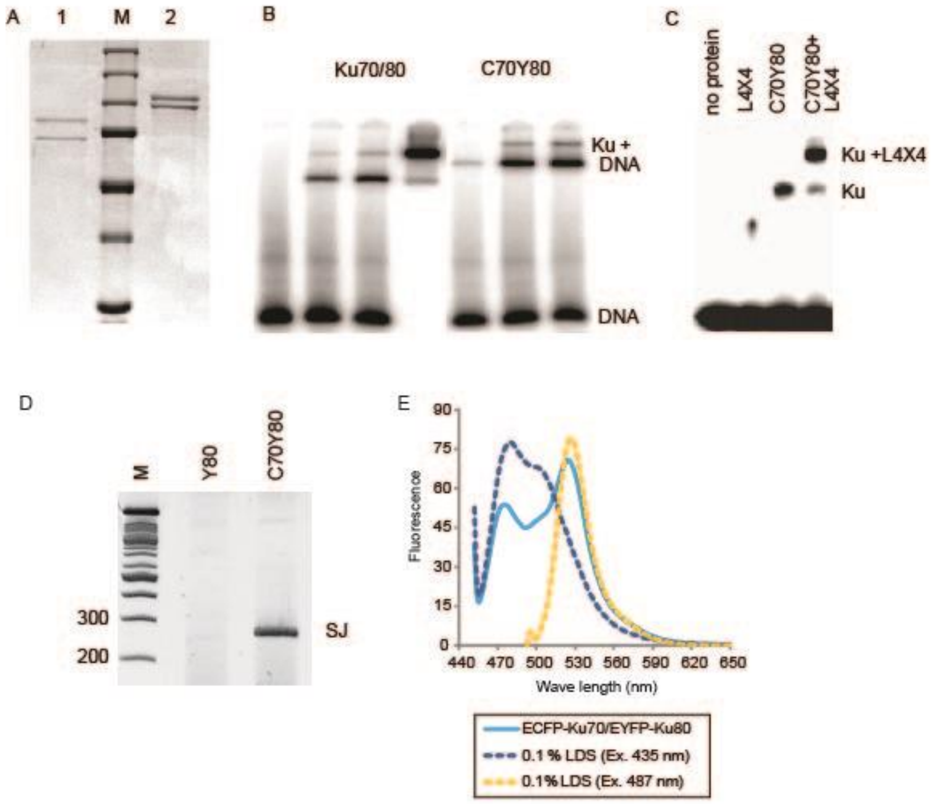

Figure S1

The ECFP-Ku70/EYFP-Ku80 FRET pair is functional. (A) SDS-PAGE analysis of purified Ku70/Ku80 (lane 1) and ECFP-Ku70/EYFP-Ku80 (C70/Y80; lane 2); M = molecular weight marker (size in kD on the left). (B) EMSA analysis of DNA end-binding capacity of Ku70/Ku80 and ECFP-Ku70/EYFP-Ku80 (from left to right: no Ku, Ku70/80 concentrations of 25, 50 and 75 ng, ECFP-Ku70/EYFP-Ku80 concentrations or 25, 50 and 75 ng) on the 50 bp double stranded oligonucleotide substrate DAR39/40. (C) EMSA analysis of a 30 bp ds oligonucleotide substrate with ECFP-Ku70/EYFP-Ku80 and/or ligase IV/XRCC4 (L4X4). (D) V(D)J recombination in Ku70$^{-/-}$/Ku80$^{-/-}$ mouse embryonic fibroblasts complemented with the expression construct pGK49 expressing EYFP-Ku80 alone (Y80) and together with the ECFP-Ku70 expression construct pHB25 (C70/Y80). Signal joint (SJ) formation in pDVG93 was determined by PCR. M = 100 bp DNA size marker, 200 and 300 bp marker bands are indicated on the left. (E) Fluorescence Emission Spectrum of ECFP-Ku70/EYFP-Ku80 (excitation at 435 nm) compared to the ECFP-Ku70 (excitation at 435 nm) and EYFP-Ku80 (excitation at 487 nm) spectra after disruption of the heterodimer with LDS.

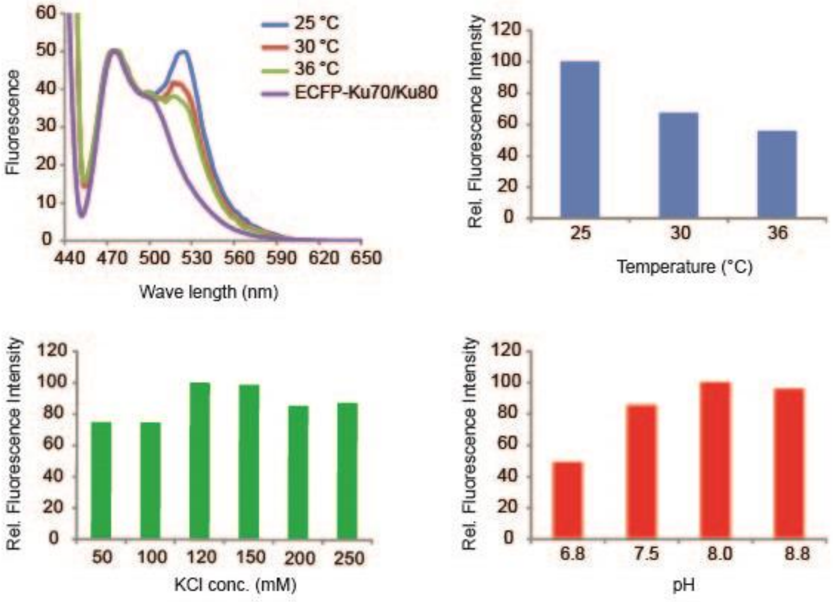

Figure S2

Optimization of the ECFP-Ku70/EYFP-Ku80 FRET signal. FRET efficiency has been calculated relative to the maximal efficiency under the most optimal conditions (120 mM KCl, pH 8.0 and 25°C).

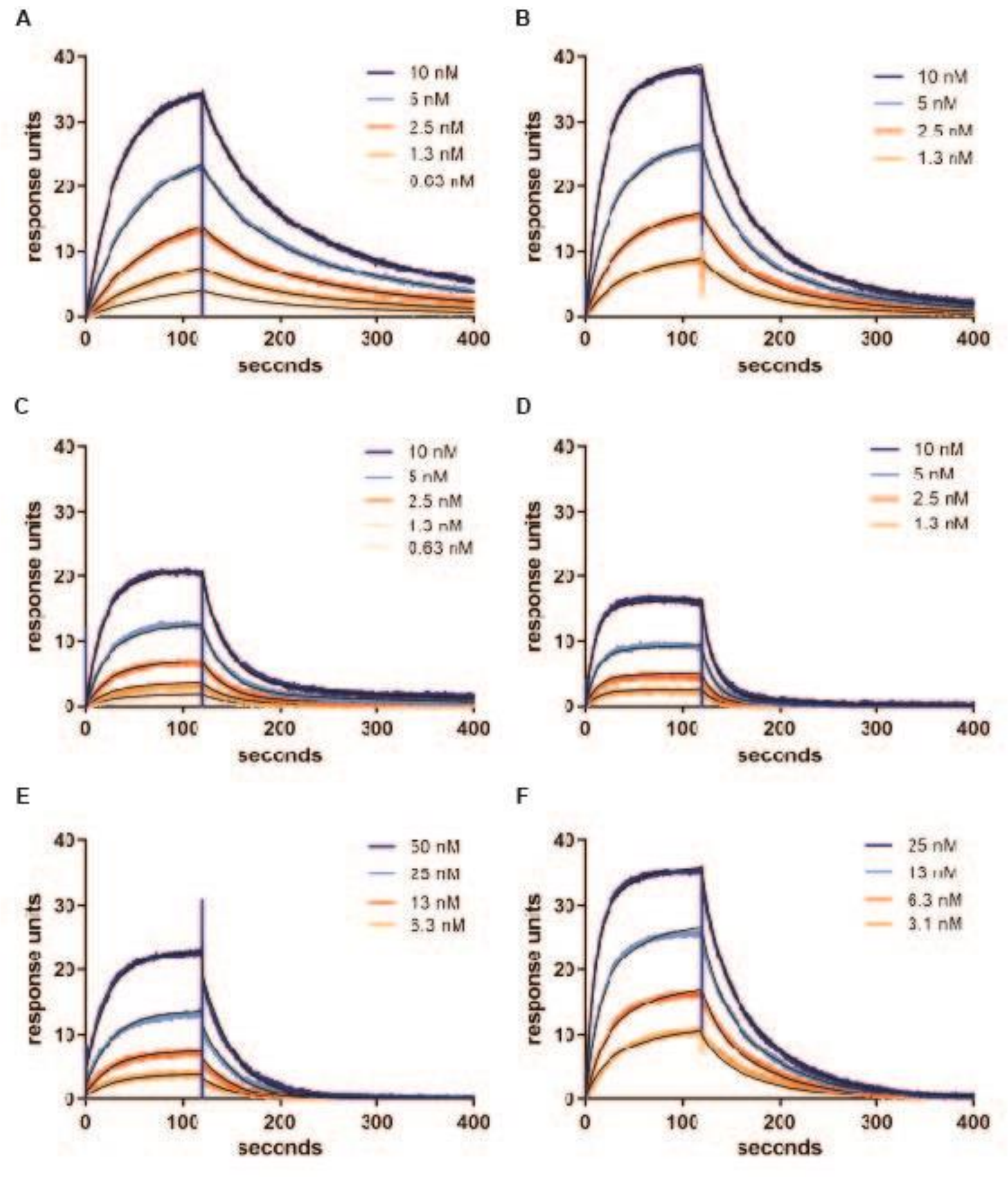

Figure S3

SPR analysis of untagged Ku, ECFP-Ku70/EYFP-Ku80, ECFP-Ku70ΔSAP/EYFP-Ku80 and ECFP-Ku70/EYFP-Ku80ΔC binding to DNA. A) Sensorgram of Ku70/80 (0.63 – 10 nM) binding to ov1 DNA at 250 mM NaCl. B) ECFP-Ku70/EYFP-Ku80 (1.3 – 10 nM) binding to ov1 DNA at 250 mM NaCl. C) Ku70/Ku80 (0.625-10 nM) binding to hp1 DNA at 250 mM NaCl. D) ECFP-Ku70/EYFP-Ku80 (1.25-10 nM) binding to hp1 DNA at 250 mM NaCl. E) ECFP-Ku70ΔSAP/EYFP-Ku80 (6.3 – 50 nM) binding to bl1 DNA. F) ECFP-Ku70/EYFP-Ku80ΔC binding to bl1 DNA (3.1 – 25 nM). Fit of the model is indicated with thin black lines.

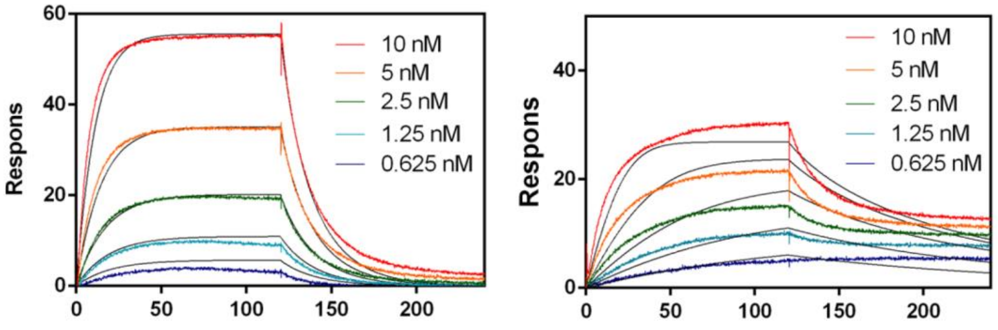

Figure S4
The effect of chip lifetime on residual binding to reference-subtracted DNA-bound flow cell. On the left residual binding on a fresh chip, with sensorgrams fitted with a 1:1 binding model. On the right residual binding of Ku on a chip surface that has been used for multiple runs, with sensorgrams fitted with a 1:1 binding model.
